# Spectrum and Antimicrobial Susceptibility Pattern of Micro-Organisms Associated With Neonatal Sepsis in a Hospital in Karachi, Pakistan

**DOI:** 10.7759/cureus.10924

**Published:** 2020-10-13

**Authors:** Mehmood Shaikh, Muhammad Hanif, Rafia Gul, Wajid Hussain, Hemandas Hemandas, Ashraf Memon

**Affiliations:** 1 Neonatology, Jinnah Sindh Medical University, Karachi, PAK; 2 Neonatology, Fatima Memorial Hospital, Lahore, PAK; 3 Paediatrics, Jinnah Sindh Medical University, Karachi, PAK; 4 Paediatrics, Peterborough City Hospital, Peterborough, GBR; 5 Pathology, Kharadar General Hospital, Karachi, PAK

**Keywords:** neonates, sepsis, causative bacteria, antimicrobial sensitivity

## Abstract

Background

Neonatal sepsis is one of the most common causes of neonatal mortality and morbidity, particularly in developing countries. Its causative bacteria and their respective sensitivity patterns are different in each hospital and region. The objective of this study was to determine the causative bacteria and their antibiotics sensitivity patterns at the neonatal unit.

Methods

This prospective study was carried out at the Neonatology Unit of Kharadar General Hospital (KGH) from January 2017 to Jun 2019. A total of 162 neonates with suspected sepsis and positive blood cultures were included in the study. Blood culture was done by standard microbiological techniques (BACTEC Method). Continuous data were presented as mean and standard deviation, while categorical data were presented in frequency and percentages.

Result

Out of a total of 162 neonates with blood culture positive neonatal sepsis, males were 106 (65.4%). Gram-positive and Gram-negative bacteria were found with a frequency of 83 (51.5%) and 79 (48.5%), respectively. Staphylococcus aureus and Pseudomonas were the commonest isolates in 50.5% and 25.7% of cases, respectively. The Gram-positive organism was mostly sensitive to amikacin and vancomycin whereas the Gram-negative was mostly sensitive to amikacin, imipenem, meropenem, and ciprofloxacin.

Conclusion

Staphylococcus aureus was the most common bacteria isolated. For the sepsis, the causative bacteria and antibiotics sensitivity pattern changes over a period of time. Continued surveillance is required to help reduce morbidity and mortality through developing institution-based guidelines.

## Introduction

Neonatal sepsis is defined as bacteremia and clinical symptoms caused by micro-organisms and their toxic products [1]. It can be either early-onset sepsis which usually presents within the first 72 hours of life or late-onset sepsis, which presents at 72 hours after birth up to 28 days of life [2].

The incidence of neonatal sepsis varies from 7.1 to 38 per 1000 live births in Asia, 6.5 to 23 per 1000 live births in Africa, 3.5 to 8.9 per 1000 live births in South America, and six to nine per 1000 live births in the United States [3]. Sepsis is a significant cause of morbidity and mortality in newborns where it contributes up to 13%-15% of deaths in developed countries, 30%-50% deaths in developing countries, and 28% deaths in Pakistan [4-6]. Clinical manifestation of neonatal sepsis is diverse which includes the maternal feeling of unwell baby, lethargy, vomiting, variability in temperature, respiratory rate and heart rate, jaundice, hepatomegaly, cyanosis, apnea, and abdominal distention [7]. In developing countries, Gram-negative organisms like Escherichia coli and Klebsiella have been found to be the leading cause of neonatal infections followed by Gram-positive organisms including Staphylococcus aureus, Group B Streptococcus (GBS), and Staphylococcus epidermidis [8-10]. In contrary to this, in developed countries, Group B Streptococcus, followed by Escherichia coli, Staphylococcus aureus, Coagulase-negative staphylococcus (CoNS), Listeria monocytogenes, Klebsiella spp., Enterococcus spp., and Pseudomonas aeruginosa are causative organisms of neonatal sepsis [11-13].

Neonatal sepsis is one of the commonest reasons for admission in the neonatology unit. As culture and sensitivity pattern varies at different places and duration, the WHO recommends determination of local culture and sensitivity pattern for starting antibiotics. In our setup, we lack the susceptibility spectrum of microorganisms responsible for neonatal sepsis, which is essential to make a choice of appropriate antibiotics in affected neonates. The results of this study will not only help to determine the causative organisms of sepsis in our population but also promote the judicious use of empirical antibiotics. This will result in shorter hospital stay of neonates, reduction in adverse effects of drugs, and cost-effective management of sepsis.

## Materials and methods

This prospective cross-sectional study was conducted from 1st January 2017 to 30th June 2019 in the neonatology unit, Kharadar General Hospital (KGH), Karachi. The study was initiated after obtaining permission from the IRB. Informed consent was obtained from the parents or guardians. A total of 162 neonates of either gender admitted in the neonatal unit through paediatric OPD/emergency room who presented with sepsis and their blood cultures were found to be positive were included in the study. Detailed history and examination were carried out in all patients presenting with sepsis. The neonates with incomplete data on proforma were excluded from the study. About 5 cc (minimum 1 cc) of venous blood samples were withdrawn and, directly injected into BACTEC® PEDS Plus (Becton Dickinson, Towson, MD) culture media bottle after using aseptic technique. The sample was transported to the laboratory for processing blood cultures. In the microbiology section of KGH lab, the BACTEC bottle was loaded in BACTEC 9050 automated blood culture instrument, based on fluorescent technology with continuous, unattended testing of cultures. When positive vials were identified, the lab technologist took out a specific bottle from the BACTEC instrument for gram staining as well as subculturing on specific media by inoculating the culture broth on blood, chocolate and MacConkey agar plates. These plates were then incubated in an incubator at 37°C in chocolate plate also under a candle jar for 24 hours. On the next day, the culture plate was read for their colony characteristic and the further biochemical test was run for the identification of specific microorganisms, as well as antibiotic susceptibility test was performed by disk diffusion technique. As per protocol, the negative sample remained the instrument for five days and then the report declared as negative. Results were analyzed using SPSS version 20 (IBM Corp., Armonk, NY). The qualitative variable was presented as mean and standard deviation, while quantitative variables presented in frequency and percentages. A Chi-square test was used to compare the variables. P-value ≤0.05 was considered statistically significant.

## Results

**Figure 1:**
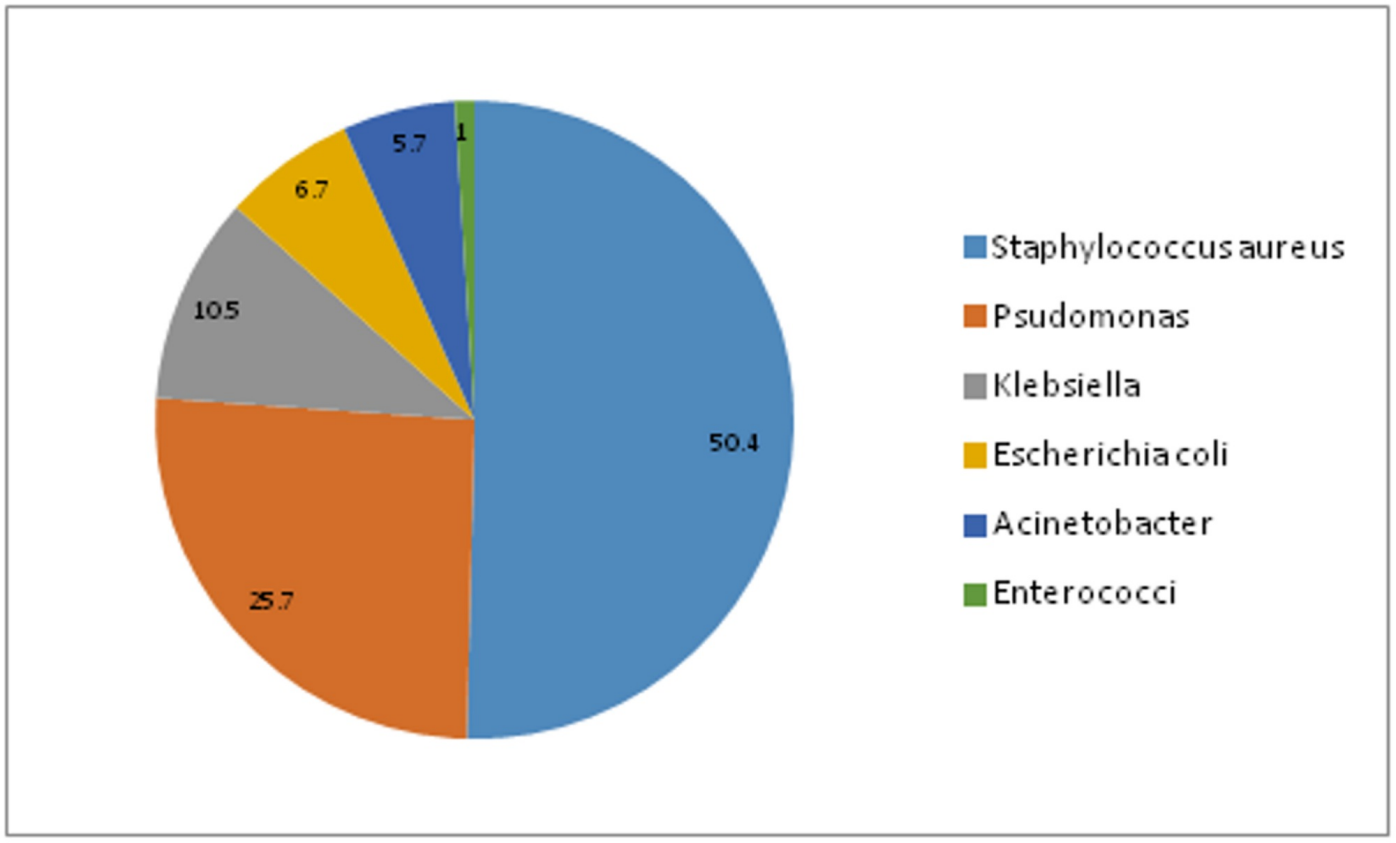
Distribution of micro-organisms involved in neonatal sepsis.

Between January 2015 and June 2017, 1960 neonates were admitted to the neonatology unit with a diagnosis of sepsis or developed sepsis during the hospital stay. A total of 162 out of 1960 neonates had a positive blood culture. A total of 106 (65.4%) were male and 56 (34.5%) were female babies making a male: female ratio 1.89:1. The mean age of admission was 140 ± 131 hours. Mean birth weight was 2.44 + 0.71 kg (0.8-4.5 kg). Gram-positive bacteria were more frequent than Gram-negative bacteria with a frequency of 83 (51.5%) and 79 (48.5%), respectively. Among Gram-positive isolates, Staphylococcus aureus (50.1%) was the commonest organism isolated followed by Enterococci spp. (1%), while in Gram-negative isolates, Pseudomonas spp. was most common (25.7%) followed by Klebsiella (10.5%) (Figure 1).

The bacteria responsible for the sepsis showed a variable pattern of antibiotics sensitivity. The results of antibiotic sensitivity, the pattern of Gram-positive bacteria were as shown in Table 1.

**Table 1:** Antibiotic sensitivity pattern of Gram-positive bacteria.

Antibiotics	Staphylococcus aureus	Enterococci spp.
Amikacin	85%	100%
Amoxicillin/clavulanate	47.1%	0%
Cefotaxime	16.9%	100%
Ceftazidime	11.3%	0%
Ceftriaxone	26.4%	100%
Chloramphenicol	68%	0%
Ciprofloxacin	16.9%	100%
Clindamycin	66%	100%
Cloxacillin	28.3%	100%
Enoxacin	47.1%	100%
Gentamicin	5.6%	0%
Lincomycin	79.24%	100%
Methicillin	15%	0%
Ofloxacin	32%	100%
Oxacillin	30.1%	100%
Vancomycin	88.67%	0%

The results of antibiotic sensitivity, the pattern of Gram-negative bacteria were as shown in Table 2.

**Table 2:** Antibiotic sensitivity pattern of Gram-negative bacteria.

Antibiotics	Pseudomonas spp.	Klebsiella spp.	Escherichia coli	Acinetobacter
Amikacin	85%	82%	100%	66.6%
Amoxicillin/Clavulanate	7.4%	36.36%	57.1%	66.6%
Cefotaxime	0%	27.27%	14.2%	0%
Ceftazidime	29.6%	9%	0%	0%
Ceftriaxone	26%	54.54%	14.2%	33.3%
Chloramphenicol	14.8%	18.1%	0%	16.6%
Ciprofloxacin	77.7%	63.6%	57.1%	16.6%
Clindamycin	7.4%	9%	0%	0%
Gentamicin	32%	63%	71.4%	66.66%
Imipenem	77.7%	100%	100%	33.3%
Meropenem	74%	100%	100%	33.3%
Ofloxacin	44.4%	9%	14.2%	0%
Oxacillin	3.7%	9%	0%	0%
Piperacillin	61.5%	0%	0%	16.6%
Tobramycin	70.3%	36.36%	42.8%	16.6%
Vancomycin	3.7%	9%	0%	0%

## Discussion

Neonatal sepsis is considered to be an important cause of neonatal morbidity and mortality [14]. Developing countries share 99% of the estimated 4 million neonatal deaths [15]. Out of 4 million one-fourth die due to neonatal sepsis [16-17]. The timely diagnosis of neonatal sepsis plays an important role in determining the outcome of the babies. Blood culture is a gold standard test for sepsis [18].

Hubacek et al. have shown that the polymorphism in genes for lysosomal binding protein is responsible for the immunological difference between males and females [19]. Marriott et al. observed that cell surface receptors like TLR 4 trigger a different response in males than females in terms of inflammatory cytokines and acute-phase reactants thus making males more prone to sepsis [20]. Our study was conducted on 162 neonates with confirmed sepsis on blood culture. Male babies were 106 (65.4%) and female babies were 56 (34.5%) with a ratio of 1.89 to 1, which is consistent with several studies [14, 21-22].

In the present study, however, 54.6% of isolates were Gram-positive bacteria while Gram-negative bacteria accounted for 45.4% of cases of neonatal septicemia. The results are consistent with other studies conducted by Draz et al. and Karlowicz et al. [17, 23]. There was a similarity in the findings in studies done by Muhammad et al., Movahedian et al., Awoniyi et al., Waseem et al., Shrestha et al., and Kayange et al.; they also found Gram-negative as the main organisms responsible for neonatal sepsis [14, 24-28]. The difference in organism patterns is because of the inclusion of hospital-acquired sepsis in our study which was not included in other studies.

Staphylococcus aureus is the most commonly isolated organism responsible for neonatal sepsis in our study. Similar results have been shown by Draz et al., Shaw et al., Mhada et al., and Najeeb et al. [17, 21, 29,30]. The other Gram-positive organisms to be isolated were Enterococci. A similar pattern of the predominance of Gram-positive organisms has been shown in studies conducted by Shrestha et al. [27]. In these studies, hospital-acquired sepsis was included.

Pseudomonas was the most common Gram-negative organism. These results are consistent with Awoniyi et al. [25]. In contrary to this, Shrestha et al. found Klebsiella pneumonia the most common Gram-negative organism [27]. Muhammad et al. and Najeeb et al. described Klebsiella in 10% of the cases [14, 30]. These findings are nearly the same as our study. Shaw et al. and Waseem et al. isolated Klebsiella as the second most commonly isolated bacteria, i.e., in 18.32% and 30% of cases, respectively [21, 26].

Staphylococcus aureus was most sensitive to amikacin and vancomycin as 85% and 88.67%, respectively. Similar results were also observed in the studies done by Shaw et al. and Najeeb et al. [21,30].

Regarding the sensitivity pattern of Pseudomonas spp., following drugs showed sensitivity in decreasing pattern as amikacin, imipenem, ciprofloxacin, meropenem, and piperacillin. A study conducted by Awoniyi et al. found Pseudomonas spp. was the most sensitive to amikacin [25]. These results coincide with our study. Pseudomonas spp. sepsis was sensitive to imipenem in 77.7% in our study which is consistent with a study done by Shrestha et al. [27]. The study performed by Shrestha et al. found that Pseudomonas showed sensitivity to ciprofloxacin (77%) which is consistent with our result [27].

Kayange et al. found Klebsiella was highly sensitive to meropenem which coincides with the results of our study [28]. In a study conducted by Najeeb et al., 93% of Klebsiella was sensitive to imipenem, 73% is sensitive to amikacin and 74% to ciprofloxin [30]. These results are in conformity with our study.

Amikacin, imipenem, meropenem, and ciprofloxacin showed a high sensitivity of 100%, 100%, 100%, and 57% in the case of E. coli. The study by Muhammad et al. found almost similar sensitivity pattern [14].

## Conclusions

Staphylococcus aureus and Pseudomonas are the most common organisms causing neonatal sepsis. Gram-positive bacteria have sensitivity to vancomycin and amikacin, while Gram-negative bacteria have sensitivity to amikacin, imipenem, meropenem, and ciprofloxacin. In our country, common pathogens responsible for neonatal sepsis change over the period of time and from region to region. Improved and revised local data on the pattern of causative bacteria and antibiotics sensitivity patterns are required to help reduce morbidity and mortality through the development of institution-based guidelines.
